# GAS2 Co-Expression Networks in Breast Cancer: Identification of Potential Biomarkers and Therapeutic Targets

**DOI:** 10.34172/apb.025.46055

**Published:** 2025-11-03

**Authors:** Samin Rahimi, Mehdi Haghi, Dariush Najarzadeh, Mohammad Ali Hosseinpour Feizi

**Affiliations:** ^1^Department of Animal Biology, Faculty of Natural Sciences, University of Tabriz, Tabriz, Iran; ^2^Faculty of Mathematics, Statistics and Computer Science, University of Tabriz, Tabriz, Iran

**Keywords:** Breast neoplasms, Co-expression, GAS-2 protein, RNA sequencing pharmacogenetics, Targeted therapy

## Abstract

**Introduction:**

Breast Cancer (BCa) remains the leading cause of cancer-related mortality among women, underscoring the need for developing more effective novel biomarkers. This study investigated the role of Growth Arrest-Specific 2 (GAS2) and its co-expressed genes in breast cancer.

**Methods:**

RNA-Seq data from 60 matched normal and malignant breast tissue samples (GSE183947) were analyzed. Expression values were normalized using FPKM, and GAS2 co-expression networks were constructed with SUM Lasso and linear regression. Gene-gene interaction networks were examined using Gephi software.

**Results:**

GAS2 expression was significantly reduced in tumors compared with normal tissues (*P*<0.01). Distinct sets of GAS2-associated genes were identified in cancer versus normal tissues, with tumor-associated partners (TAS2R14, PHF21B, CNTN5, UGT2B15) linked to drug metabolism and signaling, and normal-associated partners (DCC, STAT5A, ZCRB1) linked to transcriptional and cytoskeletal regulation. Network analysis revealed substantial differences in gene expression patterns between tumor and normal tissues, indicating GAS2’s involvement in cancer-specific signaling pathways.

**Conclusion:**

GAS2 displays context-dependent gene interactions and reduced expression in BCa, suggesting potential relevance to tumor biology. While these findings support its value as a candidate biomarker, experimental validation is required before translational applications can be established.

## Introduction

 Breast Cancer (BCa) remains the most prevalent malignancy among women all around the world, accounting for roughly one in eight female cancer diagnoses. According to global cancer data (GLOBOCAN 2020), BCa represents about 11.7% of all new cancer cases, introducing it as a leading cause of cancer-related deaths worldwide. Bio-statistically, it has been demonstrated that with a higher incidence in the developed societies, there is a disproportionately high mortality rate for patients with BCa in low- and middle-income countries, due to a limited access to early detective or therapeutic procedures.^1,2^

 Etiologically, BCa complexly arises from genetic predisposition, epigenetic alterations, as well as environmental factors, totally disrupting normal cell cycle regulation, DNA repair, and apoptosis. Regardingly, indisputable roles of genetic factors like germline mutations (including Inherited alterations, particularly in BRCA1, BRCA2, TP53, PTEN, and PALB2 that increase susceptibility to BCa), and somatic mutations (like acquired genetic changes in oncogenes (e.g., PIK3CA, HER2/ERBB2) and tumor suppressors that contribute to uncontrolled proliferation of cancerous cells) should not be underestimated. Epigenetically, DNA methylation (like aberrant hypermethylation of tumor suppressor genes (e.g., BRCA1 promoter) that silences protective pathways, while global hypomethylation can activate oncogenes), histone modifications (like altered acetylation and methylation status that influence chromatin structure and transcriptional activity, affecting genes regulating growth and apoptosis), as well as emerging of Non-coding RNAs (including microRNAs, lncRNAs that regulate the expression of oncogenes and tumor suppressors dysregulation) contributing to BCa initiation and progression.

 While, the Tumor Microenvironment (TME) of BCa plays a fundamental role, where immune cells (e.g., Tumor-Associated Macrophages (TAMs), T cells, Myeloid-Derived Suppressor Cells (MDSCs)) can either inhibit or promote the tumor progression. Immunologically, BCa frequently develop various kinds of immune evasion mechanisms, such as: PD-1/PD-L1 pathway activation (eventuating to suppression of those functional T-cell activities), secretion of immunosuppressive cytokines (e.g., IL-10, TGF-β), down-regulation of MHC molecules (leading to a reduced antigen presentation), as well as hormonal regulation (Estrogen/Progesterone Receptors (ER/PR)) and HER2 signaling (driving oncogenesis to influence the host immune interactions).

 Therapeutically, multimodal and tailored approaches have been highly recommended for managing BCa, according to molecular subtype, stage, and patient predisposition profile. They encompass surgery (lumpectomy or mastectomy with sentinel lymph node biopsy or axillary dissection), radiotherapy (as a standard post-surgery approach in many cases with BCa to reduce local recurrence), systemic therapies (like endocrine therapies (e.g., tamoxifen, aromatase inhibitors) for ER/PR-positive tumors), HER2-targeted therapies (e.g., trastuzumab, pertuzumab) for HER2-positive disease), chemotherapies for triple-negative and high-risk tumors, and immunotherapy (immune checkpoint inhibitors (e.g., anti-PD-1/PD-L1 agents) particularly in triple-negative breast cancer), as well as emerging strategies (CAR-T cells, new generation of vaccines, and combination immunotherapy approaches that are still under investigation).

 In spite of the fact that there are successful results acquired from therapeutic and diagnostic methods for patients with BCa, it can be postulated that heterogenicity in tumor biology of BCa makes the development of therapeutic or diagnostic procedures face with problematic challenges, like recurrence, emergence of primary syndromes or metastasis, as well as chemo- or radio-resistance. Hence, it seems that there is an imperative need to outstanding advances in more novel and effective molecular profiling as well as targeted immunotherapy, aimed at transforming treatment strategies, with molecular biomarkers serving as pivotal tools for BCa precision medicine.^3,4^

 Because of molecular diversity in BCa, the identification and discovery of novel biomarkers and therapeutic targets are critical for advancing early detection, prognosis, and personalized treatment strategies, ultimately improving survival and Quality Of Life (QOL). There are a wide range of rationales for it, including different subtypes of BCa with different cellular structures like Circulating Tumor Cells (CTCs), and even molecular driver and passenger mutations (leading to emergence of completely different therapeutic responses), inability of some biomarkers (ER, PR, HER2, Ki-67, PD-L1) to fully capture the complexity of the disease, as well as a high risk for developing metastasis with a developed resistance (especially in patients with triple-negative BCa). Accordingly, with a more precise look, there are several molecular signaling that have been integrated into translation medicine-based BCa therapy, leading the BCa treatment toward emerging therapeutic/diagnostic targets, aimed at tumor reprogramming (like epigenetic regulators: Histone Deacetylase Inhibitors (HDIs) and DNA methylation modulators), immune-based targets, signaling pathways (PI3K/AKT/mTOR, CDK4/6, and FGFR inhibitors, cancer stem cell markers (e.g., Notch, Wnt, Hedgehog)) may prevent recurrence and metastasis.

 Because of recent developments in high-throughput molecular-based and sequencing technology, as well as bioinformatics, BCa research has been encountering with some breakthroughs, making it possible to conduct comprehensive genomic and transcriptome profiling, as well as simplifying it to comprehend the molecular networks and immunological pathways being involved in the initial phases of tumor development, as well as their progression and their response to treatment.^5^ Among them, gene co-expression network analysis has been considered as a more popular and clinically-valuable tool for deciphering coordinated gene expression patterns and dysregulated molecular circuits, being involved in BCa heterogenicity, tumor biology, as well as drug resistance mechanisms.^6,7^

 One of those significant gene in gene co-expression network analysis is Growth Arrest-Specific 2 (GAS2), being located on chromosome 11p14.3, as a cytoskeleton-associated protein originally identified as a growth arrest–specific gene, being able to interact with actin microfilaments and microtubules, thereby stabilizing and regulating the cytoskeletal dynamics. Interestingly, GAS2 generally plays a pivotal role in the processes of cell proliferation, cell cycle regulation, apoptosis (cleavage of GAS2 through caspases during apoptosis, aimed at reorganizing the cytoskeleton), and cellular invasion. In BCa, it has been depicted that there is a dysregulated pattern of GAS2, eventuating to an enhanced invasive potential by promoting cytoskeletal flexibility and tumor cell motility, due to an overexpressed GAS2, making it as a prognosticative biomolecule or biomarker for patients with metastatic BCa.^8-10^ Additionally, it is worthy to mention that an altered GAS2 activity, being implicated in resistance to apoptosis, can contribute to BCa progression, connecting it directly to processes critical in Epithelial–Mesenchymal Transition (EMT) and metastasis. Fortunately, circulating forms of GAS2 (e.g., fragments from caspase cleavage) could potentially serve as easily-detectable non-invasive biomarkers in blood, reflecting cytoskeleton dynamicity in comparison with the other routine biomolecules. Furthermore, GAS2 provides a direct insight into metastatic potentials, which standard markers cannot fully capture it in BCa.^8,11,12^

 GAS2 may play a role in the progression of the disease. In the light of a deep need to identify trustworthy molecular biomarkers and molecular targets for improving clinical outcomes in BCa, the objective of this study is to investigate the gene co-expression networks that are associated with GAS2 in BCa and the normal tissues that are located in close proximity to them. The differential patterns of GAS2 co-expression are hypothesized to have a role in the development of BCa. Furthermore, these patterns may also identify suitable candidates for tailoring therapies and enhancing clinical management.

## Materials and Methods

###  Ethical Considerations

 There was no need for any extra ethical approval or informed consent for this study because it didn’t involve any new sample collection or experimental methods that required people to be involved in the research. The main researchers made sure that the first data collection followed the ethical rules they had set up. According to the structure and type of this study, there is no need to register for Research Ethical Committee (REC). It is worth-mentioning that all of the data supporting the findings of this study are openly available in the context of this manuscript.

###  Study Procedure

 Our RNA-Seq data named dataset GSE183947 (Platform GPL11154), being extracted from the NCBI Gene Expression Omnibus (GEO) database, was used for conducting this study. There were sixty pairs of samples in the dataset. Each pair included thirty breast tumor tissues and the proximal normal tissues, all of which were taken from the same patient.^13^

 Fragments Per Kilobase of transcript per Million mapped reads (FPKM) method was used to normalize the raw RNA-Seq data to make the results from different samples be comparable. Actually, the dataset was chosen based on a number of strict quality control standards. Our inclusion criteria were having matched tumor and normal samples and a sample size that was big enough to give strong statistical power.

 To investigate GAS2-associated gene co-expression networks in patients with BCa, advanced statistical and machine learning techniques were utilized. Initially, expression data were standardized to zero mean and unit variance using the Standard Scaler function from the scikit-learn Python package, which optimizes the performance of penalized regression models.

 The core analytical approach involved a novel extension of Lasso regression, so called SUM Lasso. This method aggregates gene selection frequencies and coefficient magnitudes over 1,000 different regularization parameter (λ) values, enhancing selection stability and reproducibility in the high-dimensional RNA-Seq context. Genes exhibiting non-zero regression coefficients in more than 50% of the iterations ( > 500 λ values) were deemed robustly co-expressed with GAS2.

###  Lasso Regression Model

 The Lasso regression model was formulated as follows:


$$\underset{\beta }{\min}(\frac{1}{2n}\sum_{i=1}^{n}{\left(Y_{i}-X_{i}\beta \right)}^{2}+\lambda \sum_{j=1}^{P}\left|\beta_{j}\right|)$$

While Y represents the expression levels of our target gene (GAS2), X denotes the expression levels of other genes, β variables introduce the regression coefficients, and λ controls the regularization strength promoting sparsity. For robust gene selection, Lasso regression was performed across 1,000 different λ values (alphas). Genes selected in more than 50% of the iterations (i.e., with non-zero coefficients in over 500 alphas) were deemed robustly co-expressed with GAS2. Furthermore, to confirm reproducibility, co-expressed genes were identified by intersecting results from multiple RNA-Seq datasets, employing pairwise, three-way, and four-way intersection analyses. Genes consistently selected across datasets were literally considered strong candidates for functional association with GAS2.

 Additionally, simple linear regression models were applied to validate gene interactions with GAS2:


$$y=\beta_{0}+\beta_{1}x_{1}+\beta_{2}x_{2}+...+\beta_{p}x_{p}+\in$$

In this formula, while Y is the expression of each gene, X is an indicator for the expression levels of GAS2, β are coefficients, and ϵ is the error term. Genes with significant *P*-values (*P* > 0.05) were considered co-expressed.

 This integrative analytical framework (combining SUM Lasso and linear regression) comprehensively provides an approach to identify more reliable gene networks associated with GAS2 expression in BCa. It is important to note that False Discovery Rate (FDR) correction using the Benjamini-Hochberg method has been applied to all of the differential expression and co-expression analyses of the selected gene. Moreover, sensitivity analyses were conducted at different thresholds (40% and 60%).

## Results

**Figure 1:**
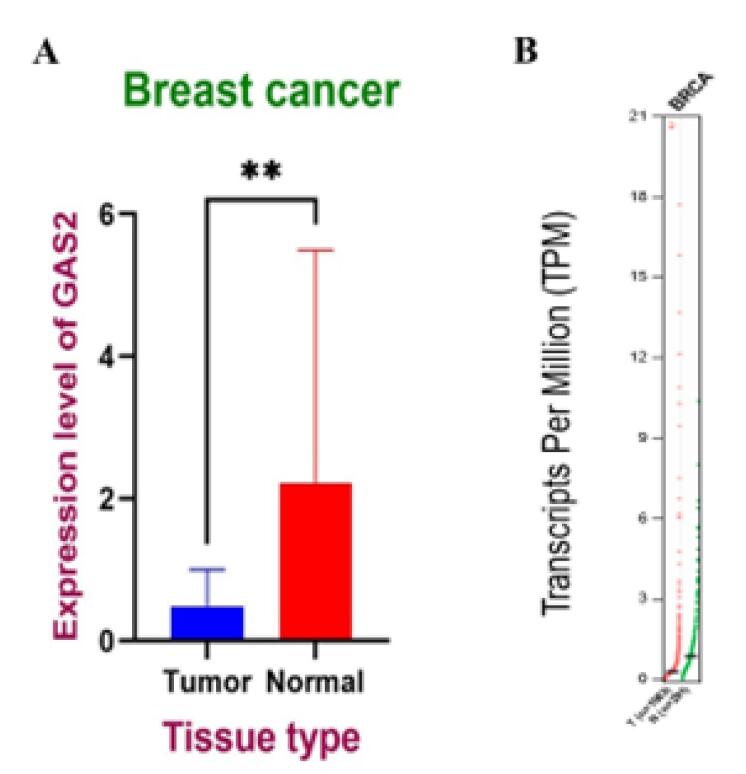
GAS2 expression (panel A = GSE183947 result; panel B = independent validation in TCGA-BRCA from GEPIA2 — TPM boxplot)

 Results from our study highlighted that the expression levels of GAS2 gene was greater in normal breast tissues when compared to those in tumor samples (*P* < 0.01) (Figure 1A). This suggests that GAS2 may play a role as a prognosticative indictor, protecting against the development of breast cancer. ^5,6^ Analysis of the broader TCGA/GTEx datasets using the GEPIA2 platform revealed that GAS2 expression levels were slightly higher in tumor samples compared to normal tissues (Figure 1B). This discrepancy in expression may be attributed to differences in data type and sample populations.

 Using linear regression and LASSO regression, some groups of genes were found that their whose expression levels were very similar to GAS2 in samples taken from normal, tumor, and mixed breast tissues (Table 1).^11,14^

**Table 1:** Genes identified via linear regression based on *P*-values.

**Normal samples**	**Tumor samples**	**Total samples**
DCC	TAS2R14	DCC
OR7C2	CNTN5	OR7C2
FRG2	RFX6	HDGFL1
HDGFL1	FAM13C	SLC34A1
SLC30A10	PHF21B	AQP12B
DACT2	CR769776.1	C1orf65
ADARB2	MGAT5B	OPN4
DACH2	PCLO	FRG2
AC012414.1	COL11A2	AC012414.1
MLC1	NPBWR2	SIAH3
SLC34A1	PVRL3	GHRHR
C1orf65	XAGE2B	DACT2
AQP12B	XAGE2	SSTR5
KRT82	NPIPA2	ADARB2
OPN4	KCNK17	KRTAP10-3
GHRHR	GNPDA2	KRT82
KRTAP10-3	GJD2	CCT8L2
SIAH3	ZNF578	CT47B1
MGAT5B	IPO11	GRM1
VWA5B2	OR52B2	IQCJ

**Figure 2:**
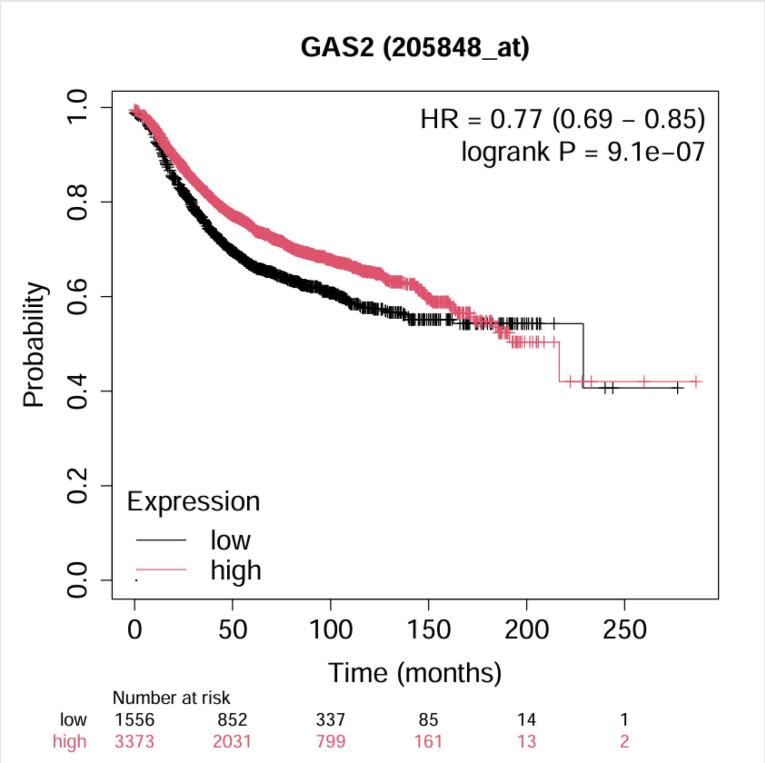
Kaplan–Meier plot (RFS) for GAS2 (KM-Plotter)

 To assess the clinical relevance of GAS2, we performed a survival analysis using the Kaplan–Meier Plotter. Patients were split into high and low GAS2 expression groups based on the optimal cutoff (34th percentile). Analysis of Relapse-Free Survival (RFS) revealed that high GAS2 expression was significantly associated with shorter RFS (median 216.66 months vs. 228.85 months for low expression; log-rank *P* = 9.1 × 10⁻⁷, FDR = 1%; Figure 2).

**Figure 3:**
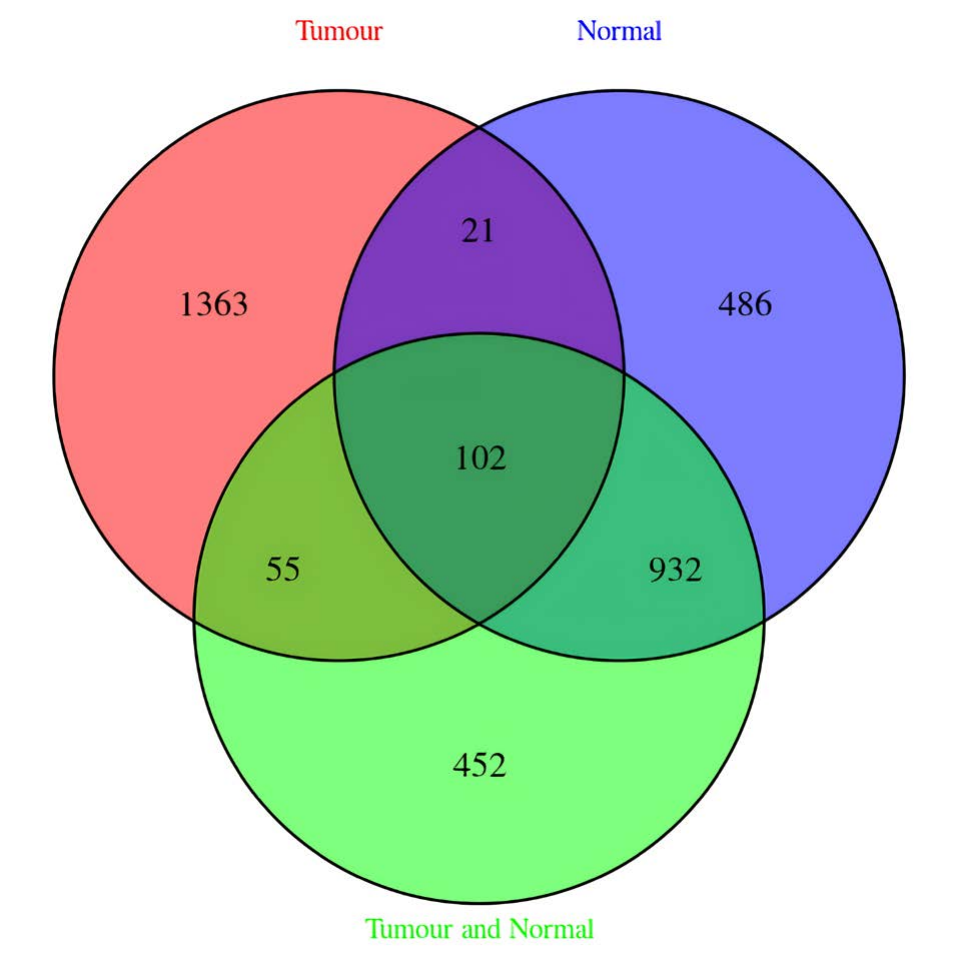
Venn diagram illustrating gene overlaps from linear regression analysis

 Specifically, on the one hand, 486 genes were found that they were only co-expressed with GAS2 in normal tissues. Many of these genes are important for keeping the cellular functions and structures in a stable mood. On the other hand, 1363 genes were found that they were only associated with GAS2 in tumor tissues. Correspondingly, these genes were often involved in cancer-related processes, like cell growth, invasion, and drug resistance.^8,15^ Also, 452 genes were found in both normal and tumor tissues, illustrating their indispensable roles in the biology of breast tissue in general. Throughout all of our analyses, a core set of 102 genes showed up over and over again. These genes probably do basic functions that are related to GAS2 (Figure 3).^13,16^

 Among the key genes, DCC, UGT2B15, and MGAT5B statistically stood out due to their strong and stable association with GAS2. In detail, DCC is known for its tumor-suppressing properties, while UGT2B15 may be involved in tumor metabolism reprogramming and drug-resistance mechanisms.^17,18^ MGAT5B is linked to changes in protein modification affecting cell signaling and adhesion, playing fundamental roles in tumor progression, and metastasis (Table 2).^9,19,20^

**Table 2:** Genes identified via linear regression based on R^2^ values.

**Normal samples**	**Tumor samples**	**Total samples**
DCC	TAS2R14	DCC
OR7C2	CNTN5	OR7C2
FRG2	RFX6	HDGFL1
HDGFL1	FAM13C	SLC34A1
SLC30A10	PHF21B	AQP12B
DACT2	CR769776.1	C1orf65
ADARB2	MGAT5B	OPN4
DACH2	PCLO	FRG2
AC012414.1	COL11A2	AC012414.1
MLC1	NPBWR2	SIAH3
SLC34A1	PVRL3	GHRHR
C1orf65	XAGE2B	DACT2
AQP12B	XAGE2	SSTR5
KRT82	NPIPA2	ADARB2
OPN4	KCNK17	KRTAP10-3
GHRHR	GNPDA2	KRT82
KRTAP10-3	GJD2	CCT8L2
SIAH3	ZNF578	CT47B1
MGAT5B	IPO11	GRM1
VWA5B2	OR52B2	IQCJ

 Tumor-specific genes such as TAS2R14 and PHF21B point to a wide array of complex molecular interactions, influencing tumor growth and monitoring responses to treatment.^8,21^ System biologically, Thyroid Stimulating Hormone Beta subunit (TSHB) conclusively emphasizes the interactions within the endocrine system in tumor development (Table 3).^22,23^

**Table 3:** Top genes obtained from LASSO analysis by cross-validation

**Normal samples**	**Tumor samples**	**Total samples**
RP11-826N14.2	TAS2R14	UGT2B15
CNTD2	PHF21B	KRT82
DCC	CNTN5	INSM1
INSM1	RFX6	DCC
BMPR1B	CR769776.1	LGI3
UGT2B15	RP11-463C8.4	FOXR1
TADA2A	TSHB	KCNT2
XKRX	UGT2B15	GHRHR
IL9R		MGAT5B
KNDC1		OR52N5
FAM83E		ZNF415
CD38		AJAP1
AC096677.1		KRT86
IL36RN		FAM151A
PMCH		SNX15
GSTM1		ZDHHC19
B3GNT4		NCAM1
ZCRB1		VAV2
CD248		CDC37L1
STAT5A		LCE2B
RPS26		GSTM1
B3GNT7		AC040160.1
ACTL8		LRRC48
WTAP		RP11-210M15.2
		SLC22A15
		FAM181B

**Figure 4:**
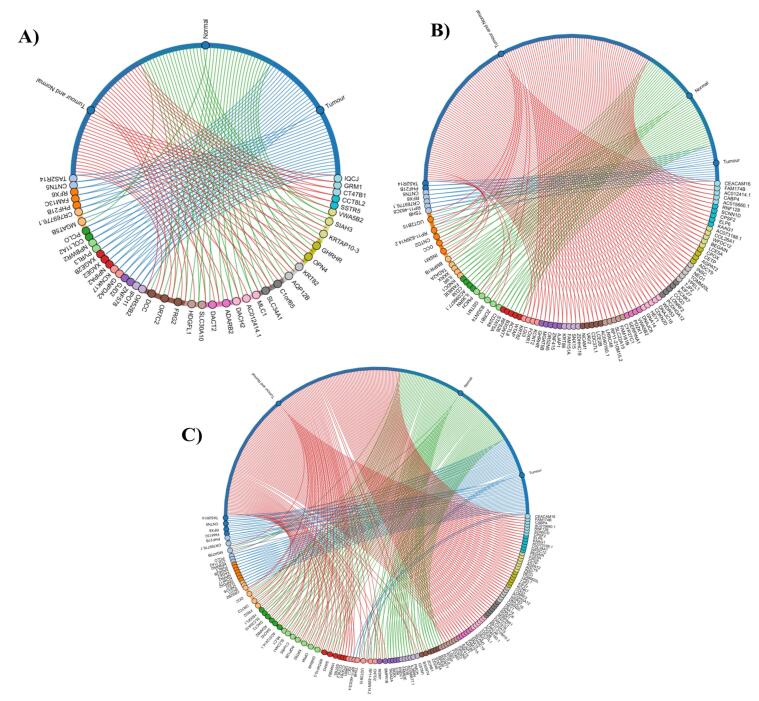
A) Combined circular chord diagram of GAS2 co-expression network B) Circular chord diagram of GAS2 co-expression network based on LASSO results C) Circular chord diagram of GAS2 co-expression network based on linear regression results

 In our study, network visualizations made it even clearer that GAS2 is a regulatory hub, with some gene interactions that are found similarly in both normal and tumor tissues, meanwhile the others were only found in one special type of tissue.^11,24,25^ This pattern hypothetically and theoretically supports the idea that GAS2 has two main functions: keeping cells functioning normally and helping cancerous cells grow (Figure 4).

 Furthermore, this collection of evidence indicates that our findings are not limited to a single method, and the results from Lasso and linear regression are consistent with the correlations and complementary analyses.

 In addition, Gene Ontology (GO) enrichment and KEGG pathway were performed. These analyses showed that the co-expressed GAS2 genes are significantly enriched in pathways related to apoptosis regulation, cytoskeletal reorganization, and hormone resistance, reinforcing the biological interpretation of our results and links the long lists of genes to specific mechanisms of BCa, paving the pathway for clinicians, oncologists, molecular biologists, gynecologists, and basic medical scientists to go through a better clinical decision, as well as efficient clinical outcomes for patients with breast cancer. All in all, these findings totally provide valuable insights into the molecular immunobiological mechanisms in BCa, involving the critical roles of GAS2, suggesting them as potential candidate targets for designing novel and more efficient therapeutic strategies, as well as clinically-acceptable diagnostic and prognosticative biomarkers for patients with BCa.

**Figure 5:**
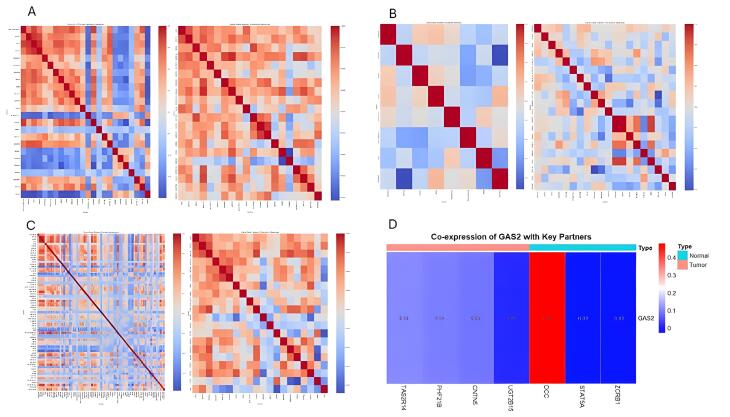
Heatmaps illustrating LASSO and linear regression results in (A) normal tissue, (B) tumor tissue, and (C) all samples. (D) Pearson correlation heatmap showing pairwise relationships between GAS2 and seven key partner genes, providing a standard correlation-based validation of the identified interactions

**Figure 6:**
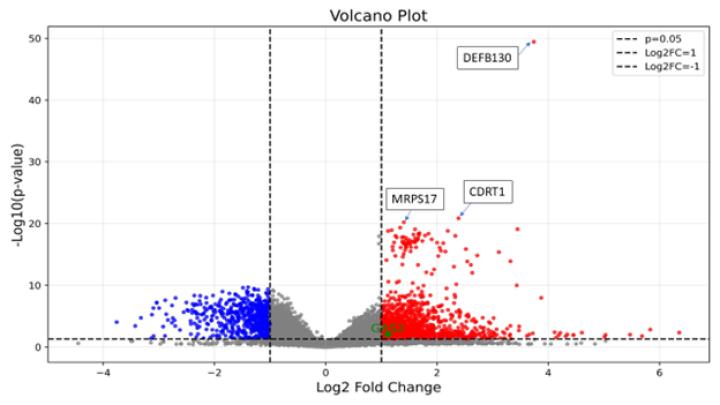
Volcano plot of differential gene expression highlighting GAS2 and tumor-specific genes

**Figure 7:**
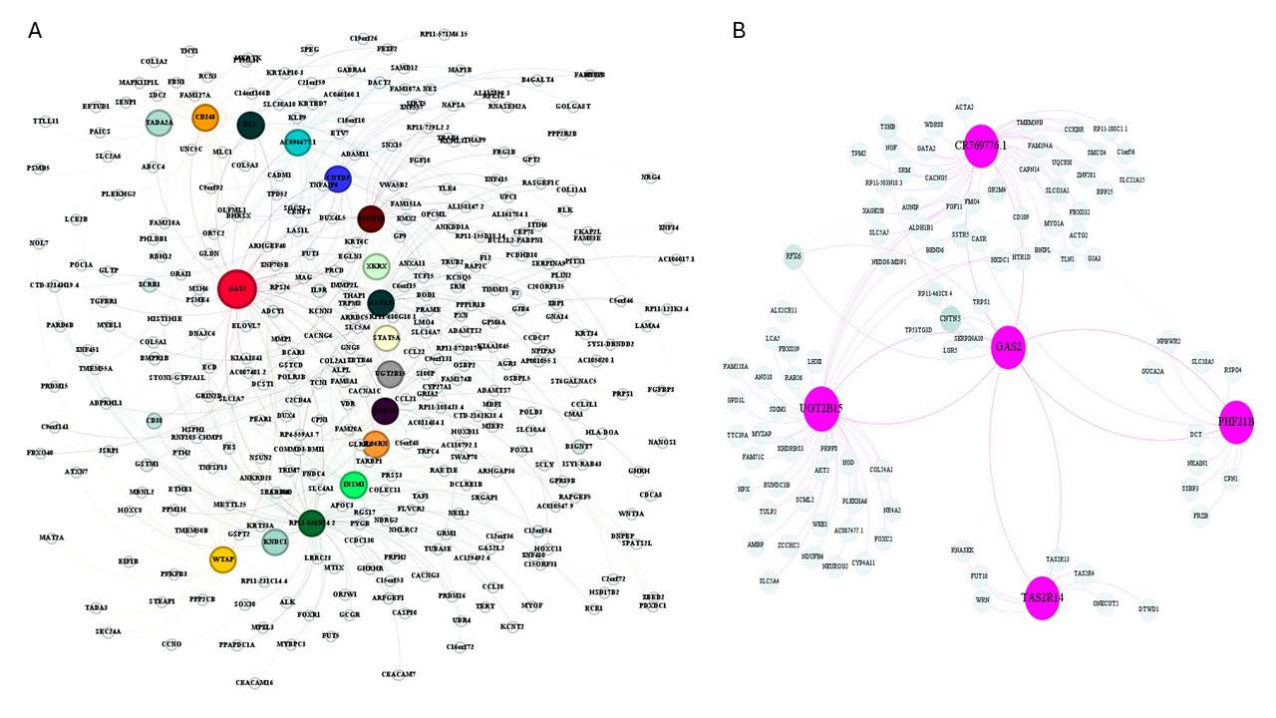
A) Gene-Gene interaction network of GAS2 in normal tissues. B) Gene-Gene interaction network of GAS2 in tumor tissues (Nodes represent genes, and edges represent gene-gene relationships)

 Furthermore, to complement and validate the LASSO and linear regression results, we performed a Pearson correlation analysis between GAS2 and its key partner genes in all samples (combined tissues). As shown in Figure 5, GAS2 displays strong positive correlations with tumor-associated partners (TAS2R14, PHF21B, CNTN5, UGT2B15) and weaker correlations with normal-associated partners (DCC, STAT5A, ZCRB1). These findings are consistent with the exploratory co-expression network analysis and provide additional support for the identified gene-gene interactions (Figure 6 and 7).

## Discussion

 According to the considerable mortality and morbidity rate of BCa, and inefficacies of current diagnostic and therapeutic approaches for some of the patients with BCa, it seems that there should be an imperative need for more highly-sensitive and efficient medical interventions for a better clinical management and targeted therapy of BCa. Moreover, high recurrence rates, risk for metastasis in an immunosuppressed TME, and resistance to therapy in patients with BCa make the oncologists, and basic medical scientists search for more efficient diagnostic and prognosticative biomarkers.^1,2^ Our study systematically examined the GAS2 co-expression network in BCa for the first time. While previous studies have linked GAS2 to cytoskeletal regulation and apoptosis, to our knowledge, no study has systematically investigated the GAS2 co-expression networks analysis in BCa. Our study uniquely identifies GAS2 interaction partners through LASSO-based network modeling, highlights potential pathways related to drug response (such as UGT2B15 and DCC), and provides a foundation for cancer translation medicine.

 In this study, expression levels of GAS2 gene, as well as co-expression levels of the other related genes and the and the genes that are turned on at the same time as it. We learned that it does a lot of different things in the cytoskeleton, like changing how cells grow and die, which is important for how well treatment works and how tumors grow.^5-7^

 We found that GAS2 expression is significantly reduced in tumor samples compared to normal breast tissue, suggesting a protective or regulatory role in normal cellular function. Through linear and LASSO regression analyses, we identified distinct sets of genes co-expressed with GAS2 specific to normal tissues, tumor tissues, and shared between both of them. Genes co-expressed with GAS2 exclusively in normal tissues such as DCC, INSM1, BMPR1B, and UGT2B15 are involved in maintaining cytoskeletal stability, apoptosis inhibition, and drug-resistance metabolism, which likely contribute to normal breast tissue homeostasis. Conversely, GAS2 in tumor tissues is associated with genes including TAS2R14, PHF21B, CNTN5, and MGAT5B, which are implicated in tumor proliferation, metastasis, and drug-resistance pathways. The presence of these tumor-specific co-expression patterns highlights GAS2’s involvement in BCa progression and therapeutic responses.

 Results from our research showed that GAS2 is an important part of networks of related genes and protein interactions that are very complicated in BCa. Previous studies have shown that caspase-3, an important apoptosis effector with enzymatic activities, has a biological effect on GAS2. It also stops calpain-2 protease from working, which refrains cytoskeletal proteins from breaking down. This means that it might be possible to change the pattern of cellular death, which can be of significance for treating cancer.^6,8^

 GAS2 does two things in different types of cancer. For example, results from another study elucidated that in hepatocellular carcinoma, GAS2 procrastinates the growth of cancerous cells by accelerating the procedures of cellular death, depending on the molecular signaling of p53.^9^ It helps some leukemic cells grow, showing a bi-functional properties in tumor biology.^7,26^ This behavior changes based on the situation, which shows how important it is to know exactly what GAS2 does at the molecular level in BCa in order to use whether its therapeutic potentials.^25^

 We used Lasso regression to find genes that are linked to tumors and that are also found with GAS2. Some of these genes are CNTN5, RFX6, TAS2R14, and PHF21B. They are involved in cellular growth, invasion, and survival.^16,21,27^ These genes could associate with GAS2 to make a worsen tumor prognosis. Not only GAS2 is linked with protective genes in normal tissues, but it also can keep the regulatory balance of the cells, which could be thrown off during the process of cancer growth.^28^

 Researchers have found UGT2B15 in both healthy and cancerous tissues, being involved in the cellular and molecular pathways that can change how breast cancer cells grow. This discovery gives us promising ideas for designing targeted therapies against BCa.^4,18^ Because it is hypothesized that it can play a role in tumors spreading, researchers need to conduct further researches to see that if GAS2-TAS2R14 axis is a good candidate for treatment of BCa.^21^ Our study also points to the possibility that chromatin remodelers like PHF21B^27^ and cell adhesion molecules like CNTN5^16^ work together to epigenetically control GAS2. These results could help the scientists make new treatments that use more than one drug. The presence of genes like MGAT5B and UGT2B15 in two chromosomal places and their different expression patterns depending on the situation^4,9^ shows how complicated GAS2-mediated networks can be, and how they respond to signals from BCa TME.

 Most importantly, GAS2 and the networks it shares with other genes are useful in medicine in ways that go beyond biology. Adding GAS2 and other genes that are similar to it to diagnostic panels could make it possible to use precision medicine strategies that are based on the unique molecular profile of each patient with BCa. This might help find patients in a proper time to have an in-time diagnosis, as well as an efficient therapeutic approach according to the genetic predisposition of each patient with BCa.^29^ Also, targeting pathways controlled by GAS2 for therapy might be able to get around resistance mechanisms, especially in cases of BCa (like triple-negative BCa) that doesn’t respond to treatment or are coping with a developed metastasis.^30^

 Previous studies have shown that GAS2 is involved in how tumors grow and how apoptosis is controlled in other cancers.^6,9^ But our computer analysis gives us new information that is only useful for BCa. Our findings indicated that in the GSE183947 dataset, GAS2 expression was higher in normal samples compared to tumor samples, whereas the analysis of TCGA/GTEx datasets revealed the opposite trend, with elevated GAS2 expression in tumors. This inconsistency may be explained by differences in data processing methods (FPKM vs. TPM), variations in sample types (paired vs. heterogeneous samples), and biological differences between adjacent normal tissues and healthy donor tissues. Consequently, these results should be regarded as exploratory, highlighting the importance of functional validation assays and multi-cohort analyses. Larger independent datasets, such as TCGA-BRCA and METABRIC, would be essential to confirm these findings, enhance statistical robustness, and ensure reproducibility and generalizability.

 Our LASSO and linear regression analyses are exploratory. To support these findings, we added a Pearson correlation analysis of key partner genes (Figure 5D), which confirms the main trends and highlights the need for further validation in independent cohorts and functional studies.

 Totally, from clinical laboratory aspects, GAS2 does not compete with the other classical biomarkers for patients with BCa, but rather adds an additional biological dimension (cytoskeletal remodeling and apoptosis regulation). When integrated into molecular profiling panels, it could enhance risk stratification, metastatic prediction, and therapy personalization for patients with BCa. As GAS2 has been proposed as a predictive biomarker for response to cytoskeleton-targeting therapies in BCa, with the potential to refine patient stratification and optimize treatment outcomes. To do these, as a prime instance, GAS2 and its key partners (UGT2B15, DCC) can be validated through qPCR and siRNA knockdown, aimed at evaluating drug sensitivity/resistance (e.g., taxane, hormone-based treatments), and survival analyses can be integrated to assess their prognostic values. In addition, from technical aspects, it is highly recommended to have the current optimal methods for normalization of data (TPM or count-based methods like DESeq2 and edgeR) in upcoming researches. Moreover, statistically, we propose that methods such as WGCNA or broader correlation analyses can provide additional insights and have been suggested as future research avenues to pave the pathway for a better analysis of this biomarker for patients with BCa. Notwithstanding the role of subtype analysis in patients with BCa that accredits the clinical decision and subsequent diagnostic-/therapeutic approaches, it can decipher the heterogeneity (e.g., ER^+^, HER2^+^) of the diseases and make a more precise measurement for these patients with a lesser confounding result. All statements regarding the potential therapeutic relevance of GAS2 have been moderated to reflect that these observations are hypothesis-generating. GAS2 and its partners may serve as potential therapeutic candidates, pending future drug screening and functional validation. We believe that These steps will enhance the translational potentials of GAS2 as a biomarker and therapeutic target in BCa translation and precision medicine.

 In summary, the key advantage of GAS2 over traditional molecular profiling markers is its direct involvement in cytoskeletal organization, invasion, and apoptosis, offering unique insight into metastatic potential and treatment resistance, especially in aggressive subtypes like triple-negative breast cancer. While ER, PR, and HER2 guide targeted therapy, GAS2 could broaden the biomarker landscape by addressing tumor invasiveness and survival mechanisms. Up to now, although GAS2 has not previously been recognized as a biomarker in BCa, our co-expression analysis reveals new associations with drug resistance and cytoskeletal pathways. This comparative context aids in understanding the position of GAS2 in the biomarker landscape.

## Conclusion

 This study comprehensively analyzed the role of GAS2 in breast cancer by examining its expression and co-expression networks in normal and tumor tissues. According to the alteration in the expression and co-expression levels of GAS2 (as a reduced expression in tumor samples) and its gene-gene interactions, it can be concluded that this gene and also its protein product can be of prominence as a predictive biomarker for patients with BCa, differential biomarkers for discriminating healthy tissues from those cancerous ones, a prognosticative one for monitoring response to treatment, as well as a promising target for tailored therapeutic strategies in BCs precision medicine and immune gene-based therapies, aimed at overcoming drug resistance and improving clinical outcomes for aforesaid patients.

## Future Perspectives

 Future research should focus on validating those molecular and protein-protein associations and unraveling the mechanistic pathways through which GAS2 influences breast cancer biology, via computer modeling and experimental validation, qPCR, immunohistochemistry, or other functional assays in independent cohort studies. Such insights are crucial for developing precision medicine approaches aimed at overcoming drug resistance and improving clinical outcomes.

 Accordingly, A major limitation of the present study is the absence of molecular subtype stratification (ER+, HER2+, TNBC). Breast cancer heterogeneity could influence gene co-expression patterns and clinical relevance of GAS2 and its partners. Therefore, our findings should be considered exploratory and hypothesis-generating. Future studies using larger cohorts with detailed subtype annotations, such as TCGA-BRCA, are necessary to validate these observations and assess subtype-specific correlations. further investigations and an interdisciplinary collaboration among the oncologists, gynecologists and obstetricians, internal medicine specialists, clinical biochemists, clinical immunologists, clinical geneticists, cellular and molecular cancer immunotherapists, clinical physiologists, cellular and molecular biologists, cellular and molecular immunobiologists, personalized medicine specialists, cellular and molecular medicine specialists, translation medicine specialists, experimental medicine specialists, cellular and molecular pharmacologists, molecular pathologists, molecular biotechnologists, medical biotechnologists, cancer biology researchers, medical laboratory scientists, basic medical scientists, biomolecule scientists, diseases-specific cellular and molecular biomarker specialists, bioinformaticians, and health system coordinators are highly recommended.

## Availability of Data and Materials

 The authors confirm that all of the data generated or analyzed during this study, supporting the findings of this study are available within this article.

## Competing Interests

 The authors declare no conflict of interest.

## Ethical Approval

 Not applicable.
